# Supplementary material for: Transforming CCTV cameras into NO2 sensors at city scale for adaptive policymaking
Source: Sci Rep. 2025 Jan 29;15:3640. doi: 10.1038/s41598-025-86532-8 (PMC11779846; doi:10.1038/s41598-025-86532-8)
Supplement: Supplementary file 1 — Supplementary Information. [file 41598_2025_86532_MOESM1_ESM.pdf]

Transforming CCTV cameras into NO_2 sensors at city scale for adaptive policymaking

Mohamed R. Ibrahim[a,b,*] and Terry Lyons[a,c]

[a]The Alan Turing Institute, London, UK.
[b]Institute for Spatial Data Science, University of Leeds, Leeds, UK.
[c]Mathematical Institute, Oxford University, Oxford, UK.
[*]Corresponding author, email: geomi@leeds.ac.uk.

Supplementary Materials

S1 - S2 Figures

S1 - S5Tables

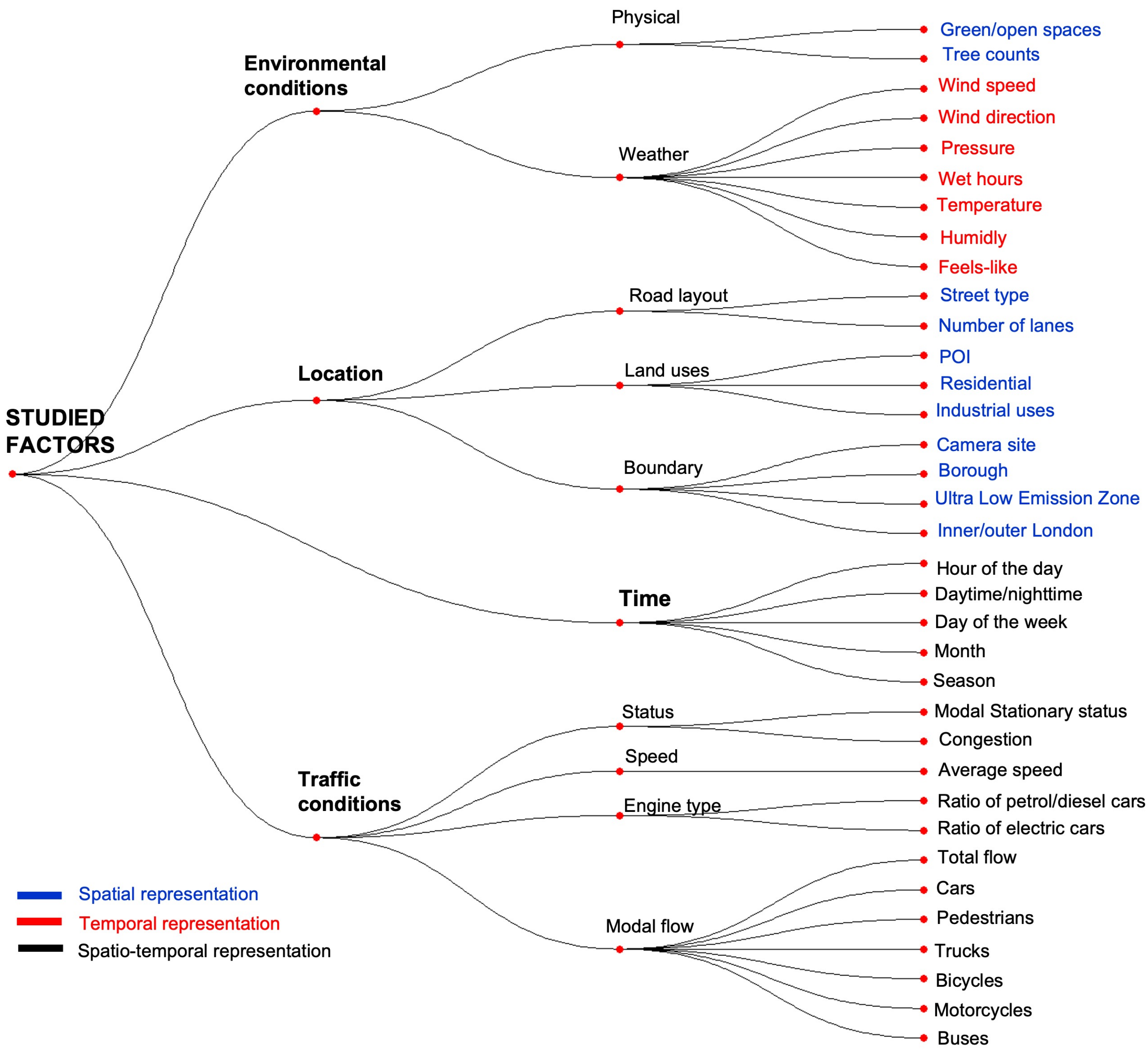

Fig. S1 Representation of key studied factors and their domain and temporal representation.

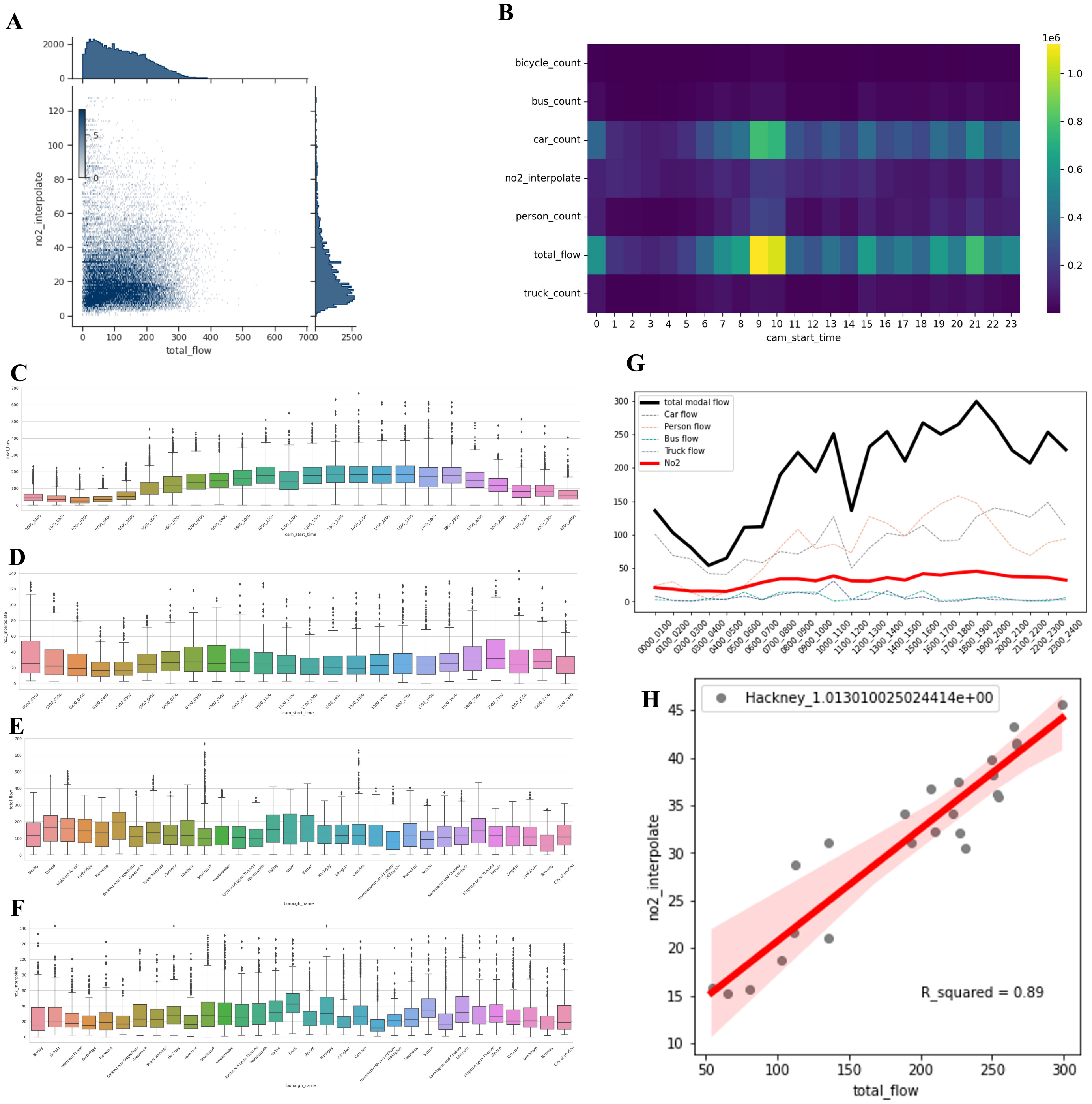

Fig. S2 Spatiotemporal patterns of traffic flows and NO_2 levels. **(A)** Relationship between NO_2 levels and total flow for each camera's location (N=88020). **(B)** A heatmap showing the relationship between the sum of a given type of traffic modes for all cameras at each hour of the day. **(C)** A box plot highlighting the distribution of total traffic at a given hour of the day, showing the morning and afternoon peaks. **(D)** A box plot highlighting the distribution of NO_2 levels at a given hour of the day. **(E)** A box plot highlighting the distribution of total traffic for each borough in London. **(F)** A box plot highlighting the distribution of NO_2 levels for each borough in London. **(G)** The sequence of the different types of traffic modes and NO_2 levels for 24 consecutive hours of a given day and location. **(H)** The relation between the total traffic modes and NO_2 levels for 24 consecutive hours of a given day and location (n=24, r^2=0.89).

Table 1 The statistics results of spatial regression model for all hours

Variable	Coeff.	z_statistics	Std. Error	P-Value
CONSTANT	-3240.8	(-26.593998485571323, 7.964633094485658e-156)	121.8621	0
car_count	0.0317	(15.17594608080966, 5.1036514079524246e-52)	0.0021	0
bus_count	0.1212	(4.968746721566333, 6.738702035838706e-07)	0.0244	0
truck_count	-0.1463	(-11.230212099503312, 2.8978208953987843e-29)	0.013	0
motorcycle_count	-0.6981	(-1.470587151254405, 0.1414028008301437)	0.4747	0.1414
car_standing	-0.0847	(-15.619868885580317, 5.331515286966446e-55)	0.0054	0
bus_standing	-0.1365	(-4.490196502306664, 7.115749769009126e-06)	0.0304	0
truck_standing	0.0578	(2.762711349797094, 0.005732343593113537)	0.0209	0.0057
motorcycle_standing	-1.4161	(-1.602778838893691, 0.1089834903414405)	0.8835	0.109
congestion	0.0607	(16.57835278251283, 9.993053353873868e-62)	0.0037	0
pressure_mean_2021	3.2438	(26.962406766252982, 4.080909118979775e-160)	0.1203	0
rainfall_2021	43.571	(30.49492834300486, 3.0422806498338705e-204)	1.4288	0
sun_hours_2021	6.4385	(39.001477963123925, 0.0)	0.1651	0
temperature_mean_2021	-2.4859	(-53.51203805390859, 0.0)	0.0465	0
wet_hours_2021	-33.7822	(-35.21633099823809, 1.1246475830658265e-271)	0.9593	0
wind_speed_mean_2021	2.8438	(19.792472757050792, 3.456596745550008e-87)	0.1437	0
Monday	-11.8181	(-10.519019132738338, 7.060475851963172e-26)	1.1235	0
Tuesday	-76.3571	(-28.311037979505937, 2.527558312459575e-176)	2.6971	0
Wednesday	-28.0184	(-17.753650712876322, 1.6150554362803476e-70)	1.5782	0
Thursday	-46.2311	(-29.82590566064918, 1.8030491978206822e-195)	1.55	0
Friday	-29.808	(-31.23690461896308, 3.3625209611926207e-214)	0.9543	0
Saturday	-45.0216	(-33.06182101991166, 1.0520591759555352e-239)	1.3617	0
proximity_industry	2.1559	(11.934337332577172, 7.838059549555e-33)	0.1806	0
wind_direction_NE	-26.8775	(-30.56339577950271, 3.7535466727372635e-205)	0.8794	0
wind_direction_SW	6.761	(3.911454462153434, 9.17419558745479e-05)	1.7285	0.0001
ultra_low_emission_zone	3.0747	(17.030505814120826, 4.878075399459055e-65)	0.1805	0
landuse_forest	1.0433	(8.045252077115027, 8.606807616698688e-16)	0.1297	0
trunk_street	1.1702	(3.5721390778755864, 0.00035407727694214850)	0.3276	0.0004
maxspeed	-0.042	(-9.303812353819943, 1.354981237316461e-20)	0.0045	0
landuse_farmland	-0.8048	(-3.223911552235775, 0.0012645244975738265)	0.2496	0.0013
W_No2	0.0224	(0.6408633874616294, 0.5216114451415428)	0.035	0.5216

Table 2: The statistics results of the spatial regression model for each hour

Hour	Variable	Coeff.	z_statistics	Std. Error	P-Value
0000-0100	CONSTANT	-17.105	(-1.422813226440614, 0.15479030611761294)	12.0219	0.1548
0000-0100	car_count	-0.6192	(-11.819739627264475, 3.0863961557362466e-32)	0.0524	0
0000-0100	car_standing	0.9992	(8.532193869768667, 1.4359814784144314e-17)	0.1171	0
0000-0100	bus_count	0.9201	(1.830824603958303, 0.06712672672985578)	0.5026	0.0671
0000-0100	bus_standing	-0.2127	(-0.3348247173472378, 0.7377573139037313)	0.6352	0.7378
0000-0100	truck_count	1.6024	(4.626597753966568, 3.717214602491084e-06)	0.3464	0
0000-0100	truck_standing	-0.5564	(-1.2998949896625933, 0.1936369625752341)	0.428	0.1936
0000-0100	congestion	-0.5106	(-5.854482869187832, 4.7849652692640265e-09)	0.0872	0
0000-0100	ultra_low_emission_zone	-1.1388	(-0.4000536583131751, 0.6891169956944563)	2.8466	0.6891
0000-0100	proximity_industry	-1.0366	(-0.5585707727513226, 0.5764546914629414)	1.8558	0.5765
0000-0100	maxspeed	0.0679	(1.986899300330918, 0.04693355718902318)	0.0342	0.0469
0000-0100	landuse_farmland	1.8319	(0.6165069992893615, 0.5375599538329356)	2.9714	0.5376
0000-0100	W_No2	1.7611	(4.98441349928278, 6.21500415990157e-07)	0.3533	0
0100-0200	CONSTANT	-50.0638	(-3.3362236447802345, 0.0008492481546687452)	15.0061	0.0008
0100-0200	car_count	-0.7554	(-11.054180078453335, 2.092399293527026e-28)	0.0683	0
0100-0200	car_standing	1.1265	(8.051222205107491, 8.197129130942354e-16)	0.1399	0
0100-0200	bus_count	2.176	(3.77490854403969, 0.0001600663777019936)	0.5764	0.0002
0100-0200	bus_standing	-1.2631	(-1.8105988663391486, 0.07020296782134858)	0.6976	0.0702
0100-0200	truck_count	1.1694	(3.3709808473980556, 0.0007490106493686409)	0.3469	0.0007
0100-0200	truck_standing	-0.3217	(-0.7274415890747786, 0.466955485158681)	0.4422	0.467
0100-0200	congestion	-0.4724	(-4.6549809145614605, 3.2401071888991335e-06)	0.1015	0
0100-0200	ultra_low_emission_zone	-8.1497	(-2.5483728599870537, 0.010822672835888104)	3.198	0.0108
0100-0200	proximity_industry	0.9368	(0.5603078041886621, 0.57526950515043)	1.6719	0.5753
0100-0200	maxspeed	0.0962	(2.607272779901439, 0.009126662120787653)	0.0369	0.0091
0100-0200	landuse_farmland	3.9429	(1.3218011607964901, 0.18623437171265134)	2.983	0.1862
0100-0200	W_No2	2.8401	(5.820974047444298, 5.850565468487407e-09)	0.4879	0
0200-0300	CONSTANT	72.6384	(6.36364438101125, 1.9702201373129983e-10)	11.4146	0
0200-0300	car_count	-0.4453	(-9.725079261257264, 2.357217449034196e-22)	0.0458	0
0200-0300	car_standing	0.5715	(6.277941271445471, 3.43085449910835e-10)	0.091	0
0200-0300	bus_count	-0.8782	(-2.1305446290287198, 0.03312667537872529)	0.4122	0.0331
0200-0300	bus_standing	1.1046	(2.182440157801496, 0.029077063275754947)	0.5061	0.0291
0200-0300	truck_count	-0.102	(-0.4883941137369355, 0.6252707104081452)	0.2089	0.6253
0200-0300	truck_standing	0.2373	(0.8295124361933988, 0.4068145026256976)	0.2861	0.4068
0200-0300	congestion	-0.3018	(-4.467035770400603, 7.931087258549044e-06)	0.0676	0
0200-0300	ultra_low_emission_zone	10.5295	(6.8481033828906535, 7.48354785962433e-12)	1.5376	0
0200-0300	proximity_industry	6.2339	(4.378411929672644, 1.1954723227246431e-05)	1.4238	0
0200-0300	maxspeed	-0.0408	(-1.6163579139991175, 0.10601695208724987)	0.0253	0.106
0200-0300	landuse_farmland	-7.0769	(-3.2923528070890624, 0.0009935288968460137)	2.1495	0.001
0200-0300	W_No2	-1.4231	(-3.276758649009386, 0.0010500608657204522)	0.4343	0.0011
0300-0400	CONSTANT	8.5543	(1.5231444169099424, 0.12772257849910715)	5.6162	0.1277
0300-0400	car_count	-0.1192	(-6.164669383684853, 7.063055580081696e-10)	0.0193	0
0300-0400	car_standing	0.1841	(3.749933225307289, 0.00017688166523440206)	0.0491	0.0002
0300-0400	bus_count	0.6259	(3.8484820542389824, 0.0001188520019062718)	0.1626	0.0001
0300-0400	bus_standing	-0.2407	(-1.1170864530977223, 0.26395736216987875)	0.2155	0.264
0300-0400	truck_count	0.6508	(6.673432185053296, 2.498890829921724e-11)	0.0975	0

Hour	Variable	Coeff.	z_statistics	Std. Error	P-Value
0300-0400	truck_standing	-0.3339	(-2.495104256216602, 0.012592012703382227)	0.1338	0.0126
0300-0400	congestion	-0.0837	(-2.0799834613307935, 0.03752704985830761)	0.0402	0.0375
0300-0400	ultra_low_emission_zone	0.8672	(1.5029560986843868, 0.1328503652424885)	0.577	0.1329
0300-0400	proximity_industry	1.2278	(1.4362871895051006, 0.15092063777272793)	0.8548	0.1509
0300-0400	maxspeed	-0.0148	(-1.1255001204793111, 0.26037716619101614)	0.0132	0.2604
0300-0400	landuse_farmland	-1.1572	(-1.077205053116908, 0.28138866830289966)	1.0742	0.2814
0300-0400	W_No2	0.572	(2.0636688398454637, 0.03904912570403052)	0.2772	0.039
0400-0500	CONSTANT	9.6552	(2.9868384013980154, 0.002818786784857815)	3.2326	0.0028
0400-0500	car_count	-0.0989	(-6.564226012647456, 5.230381902767192e-11)	0.0151	0
0400-0500	car_standing	0.168	(3.588442829265443, 0.00033265891928589377)	0.0468	0.0003
0400-0500	bus_count	0.3446	(2.8195201279255087, 0.004809551452660119)	0.1222	0.0048
0400-0500	bus_standing	0.1174	(0.7427211459173997, 0.45765052462749234)	0.158	0.4577
0400-0500	truck_count	0.6801	(9.236166425453701, 2.5548697770994693e-20)	0.0736	0
0400-0500	truck_standing	-0.3434	(-3.0118445579026685, 0.0025966552640060187)	0.114	0.0026
0400-0500	congestion	-0.0543	(-1.3758593665495125, 0.1688651773696399)	0.0394	0.1689
0400-0500	ultra_low_emission_zone	0.7408	(1.3416845271385838, 0.17969830602222148)	0.5521	0.1797
0400-0500	proximity_industry	2.2431	(2.869957297428054, 0.004105272361000629)	0.7816	0.0041
0400-0500	maxspeed	-0.0131	(-1.0250927600097801, 0.30531942136141643)	0.0128	0.3053
0400-0500	landuse_farmland	-0.5522	(-0.5404555898098896, 0.5888828799520649)	1.0218	0.5889
0400-0500	W_No2	0.4532	(2.9392039969924744, 0.0032905641526826722)	0.1542	0.0033
0500-0600	CONSTANT	1.4765	(0.41754705219623617, 0.676278315819081)	3.5362	0.6763
0500-0600	car_count	-0.0584	(-5.356501968193461, 8.484857638299245e-08)	0.0109	0
0500-0600	car_standing	0.0776	(2.058742188911834, 0.039518942375943215)	0.0377	0.0395
0500-0600	bus_count	-0.2824	(-2.9221258452879093, 0.0034765104067956193)	0.0966	0.0035
0500-0600	bus_standing	0.375	(2.8766725044951658, 0.00401892459217479)	0.1303	0.004
0500-0600	truck_count	0.1966	(3.9436237027179883, 8.025956141253998e-05)	0.0498	0.0001
0500-0600	truck_standing	-0.0772	(-0.8835682642354794, 0.3769293169499466)	0.0873	0.3769
0500-0600	congestion	0.0217	(0.6890480340196372, 0.49079304039819716)	0.0314	0.4908
0500-0600	ultra_low_emission_zone	0.0455	(0.07285989935797017, 0.9419176047687764)	0.6242	0.9419
0500-0600	proximity_industry	1.0702	(1.2479152652200547, 0.21206208971350637)	0.8576	0.2121
0500-0600	maxspeed	0.0054	(0.36430476269294887, 0.7156304437728774)	0.0147	0.7156
0500-0600	landuse_farmland	-0.3622	(-0.3098374170821315, 0.7566845960915674)	1.1691	0.7567
0500-0600	W_No2	0.9034	(6.780043086785884, 1.2014004750351273e-11)	0.1332	0
0600-0700	CONSTANT	17.9726	(3.665001893962824, 0.0002473367526108635)	4.9039	0.0002
0600-0700	car_count	-0.0284	(-2.6078568558304926, 0.009111104624793366)	0.0109	0.0091
0600-0700	car_standing	-0.0345	(-1.1223304495109225, 0.261721964116711)	0.0307	0.2617
0600-0700	bus_count	0.0771	(0.9466551813132724, 0.3438145155086414)	0.0814	0.3438
0600-0700	bus_standing	-0.1067	(-0.9695046602128293, 0.33229345618748163)	0.1101	0.3323
0600-0700	truck_count	0.108	(2.3194639230645904, 0.020369895101611815)	0.0466	0.0204
0600-0700	truck_standing	-0.0845	(-1.0545185965440356, 0.2916455510697695)	0.0801	0.2916
0600-0700	congestion	0.0986	(4.122920655142562, 3.740985875204968e-05)	0.0239	0
0600-0700	ultra_low_emission_zone	-0.1285	(-0.2114741828526926, 0.8325172757614525)	0.6076	0.8325
0600-0700	proximity_industry	2.3754	(2.7304623344912793, 0.006324555975780188)	0.87	0.0063
0600-0700	maxspeed	-0.0085	(-0.5564112327399354, 0.5779297617570607)	0.0153	0.5779
0600-0700	landuse_farmland	-1.068	(-0.8824274455016422, 0.377545699319496)	1.2102	0.3775
0600-0700	W_No2	0.3035	(1.8957234376284136, 0.057996624436640776)	0.1601	0.058
0700-0800	CONSTANT	12.8064	(2.3113166533663656, 0.02081537082798931)	5.5407	0.0208
0700-0800	car_count	0.0168	(1.5312915141914831, 0.12569736185994582)	0.011	0.1257

Hour	Variable	Coeff.	z_statistics	Std. Error	P-Value
0700-0800	car_standing	-0.1244	(-4.437282403722675, 9.110173867441231e-06)	0.028	0
0700-0800	bus_count	0.0694	(0.7660531941133752, 0.44364465155632204)	0.0906	0.4436
0700-0800	bus_standing	-0.2261	(-2.0024537015481725, 0.045235957784405444)	0.1129	0.0452
0700-0800	truck_count	0.0447	(0.9370720495236022, 0.34872149770087824)	0.0476	0.3487
0700-0800	truck_standing	-0.1031	(-1.3199612889755858, 0.18684794292495743)	0.0781	0.1868
0700-0800	congestion	0.1356	(7.017330756939118, 2.2614682950717765e-12)	0.0193	0
0700-0800	ultra_low_emission_zone	1.0992	(1.5386680830012995, 0.12388534804702023)	0.7144	0.1239
0700-0800	proximity_industry	1.6435	(1.8670231049527661, 0.06189836137860984)	0.8803	0.0619
0700-0800	maxspeed	-0.0236	(-1.3608853281221414, 0.17354993081382286)	0.0173	0.1735
0700-0800	landuse_farmland	-0.1782	(-0.13897973972306754, 0.8894661590191018)	1.2825	0.8895
0700-0800	W_No2	0.4537	(2.747752715814393, 0.00600052462852439)	0.1651	0.006
0800-0900	CONSTANT	-5.4055	(-0.9387801456674113, 0.34784363464075074)	5.758	0.3478
0800-0900	car_count	-0.0616	(-4.052649560525262, 5.064082967360317e-05)	0.0152	0.0001
0800-0900	car_standing	0.1361	(3.3412709332109403, 0.0008339580002075611)	0.0407	0.0008
0800-0900	bus_count	-0.1098	(-0.8587524741950237, 0.39047709310566614)	0.1279	0.3905
0800-0900	bus_standing	0.3701	(2.256877772684688, 0.02401571329393297)	0.164	0.024
0800-0900	truck_count	0.1853	(2.7706509455543977, 0.00559443632625586)	0.0669	0.0056
0800-0900	truck_standing	0.3434	(2.936680241670709, 0.0033174597338085344)	0.1169	0.0033
0800-0900	congestion	-0.1368	(-4.476212845534083, 7.597874091528457e-06)	0.0306	0
0800-0900	ultra_low_emission_zone	-1.4664	(-1.3070998709646098, 0.19117879511132674)	1.1219	0.1912
0800-0900	proximity_industry	0.5013	(0.4411851257607863, 0.6590789805228792)	1.1362	0.6591
0800-0900	maxspeed	0.0082	(0.3379695107089135, 0.735386164170585)	0.0244	0.7354
0800-0900	landuse_farmland	-0.2146	(-0.11609442884086187, 0.9075777041003068)	1.8489	0.9076
0800-0900	W_No2	1.2653	(7.263437860150474, 3.7737384219405366e-13)	0.1742	0
0900-1000	CONSTANT	-6.6706	(-1.3666684363326176, 0.17172925557214314)	4.8809	0.1717
0900-1000	car_count	-0.0614	(-5.048757714021111, 4.4469228379809145e-07)	0.0122	0
0900-1000	car_standing	0.1293	(4.196557903740315, 2.7100219941570485e-05)	0.0308	0
0900-1000	bus_count	-0.2764	(-2.5596030434505606, 0.010479178430431602)	0.108	0.0105
0900-1000	bus_standing	0.5356	(3.91673813898755, 8.975515074887329e-05)	0.1367	0.0001
0900-1000	truck_count	0.2637	(4.744172839407008, 2.0936003858691974e-06)	0.0556	0
0900-1000	truck_standing	0.0733	(0.8289021884558828, 0.4071597569305112)	0.0884	0.4072
0900-1000	congestion	-0.1237	(-5.728264537278641, 1.0146325948632018e-08)	0.0216	0
0900-1000	ultra_low_emission_zone	-2.4566	(-2.1521341709414576, 0.031386788515926155)	1.1415	0.0314
0900-1000	proximity_industry	0.6393	(0.6622332392391975, 0.5078217549131003)	0.9653	0.5078
0900-1000	maxspeed	0.0196	(0.9493548421218491, 0.34244016947766664)	0.0207	0.3424
0900-1000	landuse_farmland	-1.6415	(-1.0588016498164563, 0.2896901221510638)	1.5504	0.2897
0900-1000	W_No2	1.3439	(8.70569834213755, 3.156252849345412e-18)	0.1544	0
1000-1100	CONSTANT	-0.3092	(-0.08230152486511495, 0.9344069418690983)	3.7564	0.9344
1000-1100	car_count	-0.0595	(-5.103712513647672, 3.3305411184784755e-07)	0.0117	0
1000-1100	car_standing	0.126	(4.194886253925344, 2.7300855537905636e-05)	0.03	0
1000-1100	bus_count	0.1508	(1.3485831927421588, 0.17747088214903417)	0.1118	0.1775
1000-1100	bus_standing	0.0861	(0.6137524733784656, 0.5393789076303346)	0.1403	0.5394
1000-1100	truck_count	0.2977	(5.11109651069052, 3.20294295750069e-07)	0.0582	0
1000-1100	truck_standing	0.0248	(0.27266522360955026, 0.7851105695931626)	0.0909	0.7851
1000-1100	congestion	-0.1173	(-5.644581736288564, 1.6558330218078854e-08)	0.0208	0
1000-1100	ultra_low_emission_zone	-1.1356	(-1.241948144622478, 0.21425569510412268)	0.9144	0.2143
1000-1100	proximity_industry	0.4662	(0.4834725202351828, 0.6287602668798796)	0.9643	0.6288
1000-1100	maxspeed	0.0079	(0.3891923478974078, 0.6971338636137767)	0.0203	0.6971

Hour	Variable	Coeff.	z_statistics	Std. Error	P-Value
1000-1100	landuse_farmland	0.1096	(0.06942963962586732, 0.9446476368279135)	1.5787	0.9446
1000-1100	W_No2	1.1147	(8.790363555211073, 1.4907676312450971e-18)	0.1268	0
1100-1200	CONSTANT	5.7964	(1.5810402678554063, 0.11386883086647294)	3.6662	0.1139
1100-1200	car_count	-0.0274	(-2.5832917699977154, 0.00978625221458549)	0.0106	0.0098
1100-1200	car_standing	0.0291	(1.0479700029947427, 0.2946524276155944)	0.0278	0.2947
1100-1200	bus_count	0.3441	(2.8713224337877876, 0.004087583551811111)	0.1198	0.0041
1100-1200	bus_standing	-0.1679	(-1.1419501638409777, 0.253474735465701)	0.147	0.2535
1100-1200	truck_count	0.4358	(6.9098519091475294, 4.851599044974655e-12)	0.0631	0
1100-1200	truck_standing	-0.1215	(-1.2285229696970534, 0.21925071215303749)	0.0989	0.2193
1100-1200	congestion	-0.0108	(-0.5931620021394591, 0.55307273951077)	0.0183	0.5531
1100-1200	ultra_low_emission_zone	2.1103	(1.972578675196157, 0.04854358455823267)	1.0698	0.0485
1100-1200	proximity_industry	0.3068	(0.37356692833626337, 0.7087265427157636)	0.8213	0.7087
1100-1200	maxspeed	-0.0302	(-1.7383580118643138, 0.08214775030689517)	0.0174	0.0821
1100-1200	landuse_farmland	-0.2346	(-0.1721050691235831, 0.8633549276451437)	1.3634	0.8634
1100-1200	W_No2	0.6331	(4.770358795146408, 1.8389806139686912e-06)	0.1327	0
1200-1300	CONSTANT	-3.3924	(-1.4634658078082978, 0.14333996305211502)	2.318	0.1433
1200-1300	car_count	-0.0191	(-2.3953742651391434, 0.016603407735358212)	0.008	0.0166
1200-1300	car_standing	0.0386	(1.9936763482326816, 0.046187435748492114)	0.0194	0.0462
1200-1300	bus_count	-0.0265	(-0.2957731805888858, 0.7674033058827321)	0.0896	0.7674
1200-1300	bus_standing	0.114	(1.0496951228940687, 0.29385830679146263)	0.1086	0.2939
1200-1300	truck_count	0.0895	(2.0479791439056343, 0.04056204302010602)	0.0437	0.0406
1200-1300	truck_standing	0.1167	(1.7086854858126603, 0.08750922406530509)	0.0683	0.0875
1200-1300	motorcycle_count	0.4211	(1.1387970654382311, 0.25478780607461293)	0.3697	0.2548
1200-1300	motorcycle_standing	-1.2002	(-1.7183699137118276, 0.08572916446165056)	0.6985	0.0857
1200-1300	congestion	-0.0379	(-2.961420386890992, 0.003062236505885295)	0.0128	0.0031
1200-1300	ultra_low_emission_zone	-0.8467	(-1.2273614474044903, 0.2196867697715057)	0.6899	0.2197
1200-1300	proximity_industry	0.4641	(0.7071318232890271, 0.4794845613345169)	0.6563	0.4795
1200-1300	maxspeed	0.0037	(0.27200255217383623, 0.7856200569576068)	0.0135	0.7856
1200-1300	landuse_farmland	0.5362	(0.4891707854437782, 0.6247207907265608)	1.096	0.6247
1200-1300	W_No2	1.1575	(12.666143067586617, 9.10838592442768e-37)	0.0914	0
1300-1400	CONSTANT	-0.2198	(-0.06839979108685945, 0.9454673881309539)	3.2135	0.9455
1300-1400	car_count	-0.0276	(-2.8677368337176707, 0.0041341925438972225)	0.0096	0.0041
1300-1400	car_standing	0.0285	(1.2398933841673851, 0.21501483124028797)	0.023	0.215
1300-1400	bus_count	0.0111	(0.10842127313806564, 0.9136615270790855)	0.1024	0.9137
1300-1400	bus_standing	0.1285	(1.032202284271459, 0.3019773651899743)	0.1245	0.302
1300-1400	truck_count	0.2187	(4.213756179342229, 2.5115841426167324e-05)	0.0519	0
1300-1400	truck_standing	0.0325	(0.3900404077645584, 0.6965066674250593)	0.0832	0.6965
1300-1400	congestion	-0.0308	(-2.0375636974736526, 0.041593585658042)	0.0151	0.0416
1300-1400	ultra_low_emission_zone	-0.1218	(-0.14987906003154955, 0.8808600327604327)	0.8124	0.8809
1300-1400	proximity_industry	0.3506	(0.42447119551748286, 0.6712222079125278)	0.826	0.6712
1300-1400	maxspeed	-0.001	(-0.0593849739131664, 0.9526454810168881)	0.0173	0.9526
1300-1400	landuse_farmland	0.539	(0.39353531464565283, 0.6939241463458214)	1.3697	0.6939
1300-1400	W_No2	1.0066	(8.312699196446937, 9.354943568784711e-17)	0.1211	0
1400-1500	CONSTANT	-0.7632	(-0.18285576579534385, 0.8549111914045626)	4.1739	0.8549
1400-1500	car_count	-0.0134	(-1.7217208522370624, 0.0851200980900606)	0.0078	0.0851
1400-1500	car_standing	0.0136	(0.7628140410396775, 0.44557430608543647)	0.0179	0.4456
1400-1500	bus_count	0.2705	(3.138606593407384, 0.001697531821176227)	0.0862	0.0017
1400-1500	bus_standing	-0.1871	(-1.7989653574024034, 0.07202416081184737)	0.104	0.072

Hour	Variable	Coeff.	z_statistics	Std. Error	P-Value
1400-1500	truck_count	0.17	(3.739538472242519, 0.00018435843189517522)	0.0455	0.0002
1400-1500	truck_standing	0.053	(0.7220524740185159, 0.47026221630366305)	0.0734	0.4703
1400-1500	congestion	-0.0185	(-1.6100048121506372, 0.10739680576195645)	0.0115	0.1074
1400-1500	ultra_low_emission_zone	-0.1167	(-0.12873370124897857, 0.8975683675386823)	0.9064	0.8976
1400-1500	proximity_industry	0.2375	(0.3413623673705277, 0.7328308019381293)	0.6957	0.7328
1400-1500	maxspeed	-0.0155	(-1.1514698695521437, 0.24953898396970786)	0.0135	0.2495
1400-1500	landuse_farmland	0.448	(0.37976738971286506, 0.7041180912024858)	1.1798	0.7041
1400-1500	W_No2	0.9862	(5.943523942893663, 2.7895898423099583e-09)	0.1659	0
1500-1600	CONSTANT	0.089	(0.02851345030969469, 0.9772526405955085)	3.1201	0.9773
1500-1600	car_count	-0.0068	(-0.6997786286498803, 0.48406556330665207)	0.0098	0.4841
1500-1600	car_standing	-0.0299	(-1.2416377421873876, 0.21437024991724507)	0.0241	0.2144
1500-1600	bus_count	0.3122	(2.5181268450442498, 0.011798082386365505)	0.124	0.0118
1500-1600	bus_standing	-0.2818	(-1.8918961829846315, 0.058504814464961585)	0.149	0.0585
1500-1600	truck_count	0.2355	(3.559799593303441, 0.000371137913473967)	0.0661	0.0004
1500-1600	truck_standing	0.0807	(0.7911954275581424, 0.428829959316263)	0.1019	0.4288
1500-1600	congestion	0.0038	(0.23615447947094417, 0.8133128006772714)	0.0162	0.8133
1500-1600	ultra_low_emission_zone	-0.075	(-0.09126109421142045, 0.9272851312625744)	0.8219	0.9273
1500-1600	proximity_industry	0.2292	(0.2506122933029184, 0.8021138763059137)	0.9146	0.8021
1500-1600	maxspeed	-0.0129	(-0.6924173553426339, 0.4886752698470491)	0.0187	0.4887
1500-1600	landuse_farmland	0.1686	(0.11474484011885955, 0.9086473713538677)	1.4692	0.9086
1500-1600	W_No2	0.9329	(8.604338188369379, 7.675835279392488e-18)	0.1084	0
1600-1700	CONSTANT	-0.8019	(-0.174394225637206, 0.8615556520613307)	4.598	0.8616
1600-1700	car_count	0.0132	(1.4385980396276223, 0.15026444211465523)	0.0092	0.1503
1600-1700	car_standing	-0.0539	(-2.5192382519531753, 0.011760904653262108)	0.0214	0.0118
1600-1700	bus_count	0.0354	(0.30629868652826564, 0.7593772391883085)	0.1157	0.7594
1600-1700	bus_standing	0.0843	(0.5995016685302481, 0.54883839775423)	0.1406	0.5488
1600-1700	truck_count	0.3613	(4.679428788996316, 2.8767525130330154e-06)	0.0772	0
1600-1700	truck_standing	-0.0822	(-0.725978419177339, 0.46785200050772824)	0.1133	0.4679
1600-1700	congestion	0.0344	(2.4495655104317264, 0.014302868895399464)	0.014	0.0143
1600-1700	ultra_low_emission_zone	0.4722	(0.4583128394197986, 0.6467277010798711)	1.0303	0.6467
1600-1700	proximity_industry	0.4096	(0.4347159505657996, 0.6637686191140797)	0.9423	0.6638
1600-1700	maxspeed	-0.0211	(-1.1682485342554503, 0.24270652841106344)	0.018	0.2427
1600-1700	landuse_farmland	0.202	(0.14748258473519207, 0.8827511247816592)	1.3695	0.8828
1600-1700	W_No2	0.8451	(5.468728639817003, 4.532750840006737e-08)	0.1545	0
1700-1800	CONSTANT	53.1597	(7.592661935949261, 3.1339943993941106e-14)	7.0015	0
1700-1800	car_count	-0.0409	(-4.675501178143865, 2.932365936663897e-06)	0.0087	0
1700-1800	car_standing	-0.0669	(-3.007819725124115, 0.0026312917616127984)	0.0223	0.0026
1700-1800	bus_count	0.3323	(2.7896030295562655, 0.0052772702631175865)	0.1191	0.0053
1700-1800	bus_standing	-0.2328	(-1.6234636454457498, 0.10449032878954957)	0.1434	0.1045
1700-1800	truck_count	0.3243	(3.6689159845727097, 0.00024358110416401865)	0.0884	0.0002
1700-1800	truck_standing	-0.154	(-1.2377837751979737, 0.21579624603809455)	0.1244	0.2158
1700-1800	congestion	0.0444	(3.090512769921472, 0.001998112124808064)	0.0144	0.002
1700-1800	ultra_low_emission_zone	-0.3135	(-0.47998158728139223, 0.6312404857311082)	0.6532	0.6312
1700-1800	proximity_industry	4.1624	(4.977733028358603, 6.433330655400384e-07)	0.8362	0
1700-1800	maxspeed	-0.0275	(-1.6488622849540624, 0.09917585125303613)	0.0167	0.0992
1700-1800	landuse_farmland	1.1012	(0.8525393597861025, 0.3939148030444227)	1.2917	0.3939
1700-1800	W_No2	-0.8412	(-3.474864394437075, 0.0005111116551243819)	0.2421	0.0005
1800-1900	CONSTANT	9.3114	(1.7254280002318938, 0.08445036885771898)	5.3966	0.0845

Hour	Variable	Coeff.	z_statistics	Std. Error	P-Value
1800-1900	car_count	-0.0169	(-2.3915749637536403, 0.016776257639071146)	0.0071	0.0168
1800-1900	car_standing	-0.0024	(-0.13328354219144592, 0.8939691428976082)	0.0182	0.894
1800-1900	bus_count	0.3839	(4.1705471902817965, 3.0386912020756876e-05)	0.092	0
1800-1900	bus_standing	-0.2703	(-2.385699597001692, 0.01704666921397422)	0.1133	0.017
1800-1900	truck_count	0.4356	(5.812841075481985, 6.142136777492808e-09)	0.0749	0
1800-1900	truck_standing	-0.2157	(-2.062086568579402, 0.03919949487039771)	0.1046	0.0392
1800-1900	congestion	0.0091	(0.7464509267862569, 0.4553950651988873)	0.0122	0.4554
1800-1900	ultra_low_emission_zone	0.3204	(0.5404066790357264, 0.5889166027277186)	0.5929	0.5889
1800-1900	proximity_industry	1.3496	(1.7278547297278983, 0.08401427230270565)	0.7811	0.084
1800-1900	maxspeed	-0.0224	(-1.646444335189854, 0.09967231159504074)	0.0136	0.0997
1800-1900	landuse_farmland	-0.3608	(-0.328303535891946, 0.7426821709572362)	1.099	0.7427
1800-1900	W_No2	0.6129	(3.579921154115987, 0.0003436978969211541)	0.1712	0.0003
1900-2000	CONSTANT	31.0755	(4.912530276976067, 8.990849395047374e-07)	6.3258	0
1900-2000	car_count	-0.0184	(-1.4383910630621797, 0.15032312692574973)	0.0128	0.1503
1900-2000	car_standing	-0.0218	(-0.6277132353310695, 0.5301918134361415)	0.0347	0.5302
1900-2000	bus_count	0.8547	(4.953921179485579, 7.273267810405693e-07)	0.1725	0
1900-2000	bus_standing	-0.5504	(-2.4709316923382203, 0.013476155702296546)	0.2228	0.0135
1900-2000	truck_count	0.9442	(7.070584592028268, 1.5428234999852982e-12)	0.1335	0
1900-2000	truck_standing	-0.5063	(-2.6314721513160437, 0.008501583439042705)	0.1924	0.0085
1900-2000	congestion	0.027	(1.1594784795930473, 0.24626120359949777)	0.0233	0.2463
1900-2000	ultra_low_emission_zone	4.2567	(3.5236674641041845, 0.00042561804299153904)	1.208	0.0004
1900-2000	proximity_industry	4.9501	(3.602291798311308, 0.0003154240094808577)	1.3741	0.0003
1900-2000	maxspeed	-0.039	(-1.7435724787579818, 0.08123366573896032)	0.0224	0.0812
1900-2000	landuse_farmland	-3.1009	(-1.6587389951111593, 0.09716839796606679)	1.8694	0.0972
1900-2000	W_No2	-0.0921	(-0.4972835553194891, 0.6189891066113127)	0.1851	0.619
2000-2100	CONSTANT	21.7704	(3.2628926433028522, 0.0011028128769828723)	6.6721	0.0011
2000-2100	car_count	-0.0011	(-0.07906857235607473, 0.936978080853577)	0.0142	0.937
2000-2100	car_standing	-0.002	(-0.056047484277131146, 0.955303979601727)	0.0365	0.9553
2000-2100	bus_count	0.7195	(3.4170788225345476, 0.0006329695035492161)	0.2106	0.0006
2000-2100	bus_standing	-0.4378	(-1.636487180728775, 0.10173766646972411)	0.2675	0.1017
2000-2100	truck_count	0.8405	(5.397351721254542, 6.76317260873408e-08)	0.1557	0
2000-2100	truck_standing	-0.6107	(-2.8609366724401823, 0.004223914150747974)	0.2135	0.0042
2000-2100	congestion	0.0008	(0.032546273682646155, 0.9740364144954817)	0.0247	0.974
2000-2100	ultra_low_emission_zone	4.7215	(3.138990741284355, 0.0016953080427577288)	1.5041	0.0017
2000-2100	proximity_industry	3.3943	(2.548665180634531, 0.010813606344284098)	1.3318	0.0108
2000-2100	maxspeed	-0.0341	(-1.4998917835909071, 0.13364243667114942)	0.0227	0.1336
2000-2100	landuse_farmland	-0.6815	(-0.375857853326044416, 0.7070225750833969)	1.8131	0.707
2000-2100	W_No2	0.2953	(1.5893571933067356, 0.1119797731362686)	0.1858	0.112
2100-2200	CONSTANT	38.8243	(6.843206722810891, 7.743977410731256e-12)	5.6734	0
2100-2200	car_count	0.1414	(7.919058838836297, 2.3931508918998777e-15)	0.0179	0
2100-2200	car_standing	-0.228	(-4.940551198962256, 7.790203314362169e-07)	0.0462	0
2100-2200	bus_count	0.3728	(1.2786527564327461, 0.20101936318231073)	0.2916	0.201
2100-2200	bus_standing	-0.2097	(-0.5711179211033951, 0.5679197113236731)	0.3672	0.5679
2100-2200	truck_count	0.6375	(2.831888246391382, 0.00462740183264279)	0.2251	0.0046
2100-2200	truck_standing	-0.3301	(-1.1031170733211604, 0.2699763273615098)	0.2992	0.27
2100-2200	congestion	0.1505	(4.801880225194737, 1.5718270622080728e-06)	0.0313	0
2100-2200	ultra_low_emission_zone	7.1217	(5.261604416706569, 1.428037519751192e-07)	1.3535	0
2100-2200	proximity_industry	9.4025	(6.045973744272375, 1.4851028193316824e-09)	1.5552	0

Hour	Variable	Coeff.	z_statistics	Std. Error	P-Value
2100-2200	maxspeed	-0.0183	(-0.8082849163457263, 0.4189265836648054)	0.0226	0.4189
2100-2200	landuse_farmland	-1.861	(-0.989436267333816, 0.3224497375588351)	1.8809	0.3224
2100-2200	W_No2	-0.7097	(-3.563292453116102, 0.00036623227300334766)	0.1992	0.0004
2200-2300	CONSTANT	-3.3004	(-0.8042824617354022, 0.42123386413575203)	4.1035	0.4212
2200-2300	car_count	-0.0601	(-4.618812497820525, 3.859424058457775e-06)	0.013	0
2200-2300	car_standing	0.0623	(1.894252102930146, 0.05819155526750937)	0.0329	0.0582
2200-2300	bus_count	0.0248	(0.13307197906809493, 0.8941364554939463)	0.1862	0.8941
2200-2300	bus_standing	0.1058	(0.4668347550738804, 0.6406181080269093)	0.2267	0.6406
2200-2300	truck_count	0.4878	(3.4412887497968527, 0.0005789503482378232)	0.1417	0.0006
2200-2300	truck_standing	-0.3641	(-1.8846008395213232, 0.0594837488895432)	0.1932	0.0595
2200-2300	congestion	-0.0444	(-1.9893272148625123, 0.046665097663287865)	0.0223	0.0467
2200-2300	ultra_low_emission_zone	-0.1148	(-0.15518557036540953, 0.8766750236561937)	0.7396	0.8767
2200-2300	proximity_industry	-0.0947	(-0.10511146144970214, 0.916287364764925)	0.9009	0.9163
2200-2300	maxspeed	0.0068	(0.4389055671089385, 0.660729962700175)	0.0156	0.6607
2200-2300	landuse_farmland	0.4318	(0.33018998541754424, 0.7412564133809527)	1.3078	0.7413
2200-2300	W_No2	1.175	(8.833442354179356, 1.0150265451114971e-18)	0.133	0
2300-2400	CONSTANT	2.0293	(0.5826549420596769, 0.5601256112396158)	3.4828	0.5601
2300-2400	car_count	-0.0215	(-1.6503829076147054, 0.09886464476727462)	0.013	0.0989
2300-2400	car_standing	-0.01	(-0.2813644550450018, 0.7784308780752109)	0.0354	0.7784
2300-2400	bus_count	-0.0029	(-0.01753820282184784, 0.98600725608255)	0.165	0.986
2300-2400	bus_standing	-0.0381	(-0.1774146984156262, 0.8591826627874374)	0.2147	0.8592
2300-2400	truck_count	0.1764	(1.3614764108283581, 0.17336318322166921)	0.1296	0.1734
2300-2400	truck_standing	-0.032	(-0.1801585450564471, 0.8570281021224445)	0.1777	0.857
2300-2400	congestion	0.0181	(0.7021424233338999, 0.48259034800831413)	0.0258	0.4826
2300-2400	ultra_low_emission_zone	0.4688	(0.7248228923302461, 0.4685606898228226)	0.6468	0.4686
2300-2400	proximity_industry	0.6385	(0.8773662325801423, 0.3802877480286384)	0.7278	0.3803
2300-2400	maxspeed	0.0065	(0.48301920222555284, 0.6290821019619306)	0.0134	0.6291
2300-2400	landuse_farmland	-0.0494	(-0.04354357043845299, 0.9652682332560485)	1.1344	0.9653
2300-2400	W_No2	0.9075	(6.060605791634237, 1.3560982202380852e-09)	0.1497	0

Table 3 Results of Granger causality analysis for all factors in relation to NO_2 levels. Symbols for tests: α: SSR F-test, β: SSR Chi^2 test, γ: Likelihood ratio test, δ: Parameter F-test

Variable	CAM ID	Tests	Lag 1	Lag 2	Lag 3	Lag 4	Lag 5
Car count	Hackney – 1.01301002	α	0.0521 (p0.8219)	2.2857 (p0.1339)	1.5208 (p0.2559)	3.5639 (p0.0469)	2.5550 (p0.1267)
	(index=2)	β	0.0603 (p0.8060)	5.9998 (p0.0498)	7.0189 (p0.0713)	27.0857 (p0.0000)	32.8494 (p0.0000)
		γ	0.0603 (p0.8061)	5.2775 (p0.0715)	6.0161 (p0.1108)	16.8352 (p0.0021)	18.6929 (p0.0022)
		δ	0.0521 (p0.8219)	2.2857 (p0.1339)	1.5208 (p0.2559)	3.5639 (p0.0469)	2.5550 (p0.1267)
Truck (standing)	Hackney – 1.01301002	α	6.0843 (p0.0233)	2.5576 (p0.1087)	1.3949 (p0.2886)	1.0889 (p0.4128)	1.2504 (p0.3794)
	(index=2)	β	7.0450 (p0.0079)	6.7137 (p0.0348)	6.4382 (p0.0921)	8.2756 (p0.0820)	16.0772 (p0.0066)
		γ	6.1117 (p0.0134)	5.8255 (p0.0543)	5.5816 (p0.1338)	6.8695 (p0.1429)	11.4886 (p0.0425)
		δ	6.0843 (p0.0233)	2.5576 (p0.1087)	1.3949 (p0.2886)	1.0889 (p0.4128)	1.2504 (p0.3794)
Truck (standing)	Hackney-1.01406002	α	6.1217 (p0.0230)	5.4456 (p0.0157)	3.6537 (p0.0415)	1.5187 (p0.2692)	1.0317 (p0.4669)
	(index=8)	β	7.0883 (p0.0078)	14.2946 (p0.0008)	16.8632 (p0.0008)	11.5418 (p0.0211)	13.2642 (p0.0210)
		γ	6.1445 (p0.0132)	10.9034 (p0.0043)	12.2296 (p0.0066)	9.0185 (p0.0606)	9.9378 (p0.0770)
		δ	6.1217 (p0.0230)	5.4456 (p0.0157)	3.6537 (p0.0415)	1.5187 (p0.2692)	1.0317 (p0.4669)
Truck count	Hackney – 1.01403999	α	6.4913 (p0.0197)	3.9238 (p0.0411)	3.4478 (p0.0486)	4.0535 (p0.0331)	3.4907 (p0.0669)
	(index=7)	β	7.5162 (p0.0061)	10.3000 (p0.0058)	15.9129 (p0.0012)	30.8069 (p0.0000)	44.8799 (p0.0000)
		γ	6.4657 (p0.0110)	8.3810 (p0.0151)	11.7073 (p0.0085)	18.3106 (p0.0011)	22.5154 (p0.0004)
		δ	6.4913 (p0.0197)	3.9238 (p0.0411)	3.4478 (p0.0486)	4.0535 (p0.0331)	3.4907 (p0.0669)
Truck count	Hackney – 1.014089941	α	0.1311 (p0.7213)	0.4454 (p0.6483)	1.5225 (p0.2554)	2.6202 (p0.0988)	4.3103 (p0.0413)
	(index=11)	β	0.1518 (p0.6968)	1.1693 (p0.5573)	7.0267 (p0.0711)	19.9132 (p0.0005)	55.4185 (p0.0000)
		γ	0.1513 (p0.6973)	1.1379 (p0.5661)	6.0219 (p0.1106)	13.6210 (p0.0086)	25.3045 (p0.0001)
		δ	0.1311 (p0.7213)	0.4454 (p0.6483)	1.5225 (p0.2554)	2.6202 (p0.0988)	4.3103 (p0.0413)
Bus (standing)	Richmond – 1.0662499	α	13.2407 (p0.0017)	6.7214 (p0.0076)	4.7316 (p0.0192)	4.2102 (p0.0297)	1.9518 (p0.2037)
	(index=420)	β	15.3313 (p0.0001)	17.6437 (p0.0001)	21.8383 (p0.0001)	31.9977 (p0.0000)	25.0941 (p0.0001)
		γ	11.6334 (p0.0006)	12.8071 (p0.0017)	14.7616 (p0.0020)	18.7595 (p0.0009)	15.7143 (p0.0077)
		δ	13.2407 (p0.0017)	6.7214 (p0.0076)	4.7316 (p0.0192)	4.2102 (p0.0297)	1.9518 (p0.2037)
Bus count	Hackney – 1.014070034	α	2.1090 (p0.1628)	1.5828 (p0.2359)	1.6537 (p0.2256)	1.6120 (p0.2459)	6.4287 (p0.0150)
	(index=9)	β	2.4420 (p0.1181)	4.1550 (p0.1252)	7.6323 (p0.0543)	12.2511 (p0.0156)	82.6544 (p0.0000)
		γ	2.3157 (p0.1281)	3.7912 (p0.1502)	6.4650 (p0.0911)	9.4547 (p0.0507)	30.9838 (p0.0000)
		δ	2.1090 (p0.1628)	1.5828 (p0.2359)	1.6537 (p0.2256)	1.6120 (p0.2459)	6.4287 (p0.0150)
Bus count	Hackney – 1.0141400098	α	12.1979 (p0.0024)	6.2016 (p0.0101)	6.2687 (p0.0073)	3.0672 (p0.0686)	4.7238 (p0.0331)
	(index=15)	β	14.1239 (p0.0002)	16.2792 (p0.0003)	28.9323 (p0.0000)	23.3110 (p0.0001)	60.7350 (p0.0000)
		γ	10.9101 (p0.0010)	12.0522 (p0.0024)	17.8941 (p0.0005)	15.2115 (p0.0043)	26.5629 (p0.0001)
		δ	12.1979 (p0.0024)	6.2016 (p0.0101)	6.2687 (p0.0073)	3.0672 (p0.0686)	4.7238 (p0.0331)
Congestion	Hackney – 1.01301002	α	2.0070 (p0.1728)	1.5517 (p0.2421)	1.2760 (p0.3237)	1.8226 (p0.2011)	7.1196 (p0.0114)
	(index=2)	β	2.3239 (p0.1274)	4.0732 (p0.1305)	5.8893 (p0.1171)	13.8517 (p0.0078)	91.5375 (p0.0000)
		γ	2.2092 (p0.1372)	3.7228 (p0.1555)	5.1620 (p0.1603)	10.4037 (p0.0341)	32.5061 (p0.0000)
		δ	2.0070 (p0.1728)	1.5517 (p0.2421)	1.2760 (p0.3237)	1.8226 (p0.2011)	7.1196 (p0.0114)

Table 4 The results of the t-test models for all factors grouped by whether the samples are outside or inside the ULEZ. Test 1: NO_2 outside and inside ULEZ, Test 2: Truck flow outside and in the ULEZ zone, Test 3: Bus flow outside and in the ULEZ zone, Test 4: Car flow outside and in the ULEZ zone, Test 5: Bicycle flow outside and in the ULEZ zone

**Independent t-test**	**Test 1**	**Test 2**	**Test 3**	**Test 4**	**Test 5**
**Difference (outside-inside the zone)**	-3.7439	2.22	-3.1366	25.1771	-1.2931
**Degrees of freedom**	88020	88020	88020	88020	88020
**t**	-25.7615	31.6335	-58.9364	59.0456	-54.5541
**Two side test p value**	0	0	0	0	0
**Difference ¡ 0 p value**	0	1	0	1	0
**Difference ¿ 0 p value**	1	0	1	0	1
**Cohen's d**	-0.1748	0.2146	-0.3999	0.4006	-0.3701
**Hedge's g**	-0.1748	0.2146	-0.3999	0.4006	-0.3701
**Glass's delta1**	-0.1821	0.1948	-0.495	0.3489	-0.5017
**Point-Biserial r**	-0.0865	0.106	-0.1948	0.1952	-0.1808

Table 5 The results of trained models for inferring NO_2 levels. All results are shown for the validation set. All models are trained using Nvidia RTX A5000-16GB, CPU Xeon -12 cores, Ram 128G. Architectures: A: Graph Convolutional model, B: Attention-based Graph model, C: Transformer model, D: Conv1D-based multiple branch model. Task 1 represents NO_2 detection at a given location and Task 2 represents NO_2 detection at city-wide.

Task	Model ID	Signature (N)	Edge	Architecture	Params	Train time (h)	MSLE	MAE	MSE	Kl-divergence	R2
Task 1	1	Signature (N=3)	KNN - 10	A	55,839	6.4	0.0375	0.6558	0.6558	-	0.41
	2	No Signature	KNN - 10	A	55,839	6.4	0.0281	0.5722	0.5722	-	0.21
	3	No Signature	KNN - 10	B	120,342,324	5.15	0.0454	0.6842	0.6930	-	0.40
	4	Signature (N=3)	KNN - 10	B	120,342,324	5.15	0.0517	0.7249	0.9386	-	0.34
TASK 2	5	No Signature	KNN - 10	C	8,258,720	0.6	0.0526	0.7313	0.8097	0.0044	0.39
	6	No Signature	KNN - 50	C	8,258,720	0.6	0.0374	0.6019	0.5516	0.0032	0.35
	7	Signature (N=3)	KNN - 10	C	164,498,712	2.16	0.0498	0.7007	0.7511	0.0005	0.65
	8	Signature (N=3)	KNN - 50	C	164,498,712	2.16	0.0479	0.6836	0.7360	0.0004	0.73
	9	No Signature	KNN - 10	D	6,367,866	0.6	0.0380	0.6109	0.6444	0.0004	0.75
	10	No Signature	KNN - 20	D	6,367,866	0.6	0.0337	0.5751	0.5053	0.0003	0.79
	11	No Signature	KNN - 30	D	6,367,866	0.6	0.0370	0.6118	0.6012	0.0041	0.76
	12	No Signature	KNN - 40	D)	6,367,866	0.6	0.0309	0.5524	0.4752	0.0018	0.79
	13	No Signature	KNN - 50	D	6,367,866	0.6	0.0337	0.5668	0.5319	0.0181	0.78
	14	No Signature	KNN - 60	D	6,367,866	0.6	0.0331	0.5723	0.5125	0.0125	0.78
	15	Signature (N=3)	KNN - 10	D	7,909,588	0.8	0.0345	0.5782	0.5741	0.0003	0.75
	16	Signature (N=3)	KNN - 20	D	7,909,588	0.8	0.0308	0.5503	0.4644	0.00025	0.79
	17	Signature (N=3)	KNN - 30	D	7,909,588	0.8	0.0346	0.5842	0.550	0.0284	0.77
	18	Signature (N=3)	KNN - 40	D	7,909,588	0.8	0.0299	0.5426	0.4508	0.00001	0.81
	19	Signature (N=3)	KNN - 50	D	7,909,588	0.8	0.0285	0.5197	0.4251	0.00001	0.82
	20	Signature (N=3)	KNN - 60	D	7,909,588	0.8	0.0294	0.5377	0.4452	0.00001	0.82
